# Closed-Loop Versus Conventional Mechanical Ventilation in COVID-19 ARDS

**DOI:** 10.1177/08850666211024139

**Published:** 2021-10

**Authors:** Pedro David Wendel Garcia, Daniel Andrea Hofmaenner, Silvio D. Brugger, Claudio T. Acevedo, Jan Bartussek, Giovanni Camen, Patrick Raphael Bader, Gregor Bruellmann, Johannes Kattner, Christoph Ganter, Reto Andreas Schuepbach, Philipp Karl Buehler

**Affiliations:** 1 Institute of Intensive Care Medicine, 27243 University Hospital of Zurich, Zurich, Switzerland; 2 Division of Infectious Diseases, 27243 University Hospital of Zurich, University of Zurich, Zurich, Switzerland

**Keywords:** COVID-19, pandemic, acute respiratory distress syndrome, mechanical ventilation, closed loop ventilation, Intellivent, lung protective ventilation

## Abstract

**Background:**

Lung-protective ventilation is key in bridging patients suffering from COVID-19 acute respiratory distress syndrome (ARDS) to recovery. However, resource and personnel limitations during pandemics complicate the implementation of lung-protective protocols. Automated ventilation modes may prove decisive in these settings enabling higher degrees of lung-protective ventilation than conventional modes.

**Method:**

Prospective study at a Swiss university hospital. Critically ill, mechanically ventilated COVID-19 ARDS patients were allocated, by study-blinded coordinating staff, to either closed-loop or conventional mechanical ventilation, based on mechanical ventilator availability. Primary outcome was the overall achieved percentage of lung-protective ventilation in closed-loop versus conventional mechanical ventilation, assessed minute-by-minute, during the initial 7 days and overall mechanical ventilation time. Lung-protective ventilation was defined as the combined target of tidal volume <8 ml per kg of ideal body weight, dynamic driving pressure <15 cmH_2_O, peak pressure <30 cmH_2_O, peripheral oxygen saturation ≥88% and dynamic mechanical power <17 J/min.

**Results:**

Forty COVID-19 ARDS patients, accounting for 1,048,630 minutes (728 days) of cumulative mechanical ventilation, allocated to either closed-loop (n = 23) or conventional ventilation (n = 17), presenting with a median paO_2_/ FiO_2_ ratio of 92 [72-147] mmHg and a static compliance of 18 [11-25] ml/cmH_2_O, were mechanically ventilated for 11 [4-25] days and had a 28-day mortality rate of 20%. During the initial 7 days of mechanical ventilation, patients in the closed-loop group were ventilated lung-protectively for 65% of the time versus 38% in the conventional group (Odds Ratio, 1.79; 95% CI, 1.76-1.82; *P* < 0.001) and for 45% versus 33% of overall mechanical ventilation time (Odds Ratio, 1.22; 95% CI, 1.21-1.23; *P* < 0.001).

**Conclusion:**

Among critically ill, mechanically ventilated COVID-19 ARDS patients during an early highpoint of the pandemic, mechanical ventilation using a closed-loop mode was associated with a higher degree of lung-protective ventilation than was conventional mechanical ventilation.

## Background

Coronavirus disease 2019 (COVID-19) triggered a surge of critically ill patients with acute respiratory distress syndrome (ARDS) in need of mechanical ventilation.^1^ Optimal management of ARDS mandates lung-protective mechanical ventilation so as to minimize ventilator induced lung injury (VILI) and allow for optimal recovery of the lung.^2–4^ Due to the high number of patients, intensive care units (ICUs) worldwide have been overwhelmed, leading to a shortage in the expertise and resources needed to ensure the implementation of such lung-protective settings.^5–7^ Consequently, the incidence of VILI has risen markedly and mortalities in COVID-19 ARDS (CARDS) are reaching levels not experienced for decades in the setting of ARDS.^8–10^

In critical care, the implementation of tools that reduce the strain on nursing and medical staff, while offering equal or better benefit to the patient, may turn out to be decisive in such a resource-intensive disease as CARDS. Closed-loop mechanical ventilation could help clinicians in the systematic implementation of lung-protective ventilation in CARDS patients, while significantly reducing their workload.

Closed-loop mechanical ventilation modes enable a fully automatized and optimized function of the mechanical ventilator, thus reducing the necessity for manual adjustment.^11^ INTELLiVENT®-ASV is such a closed-loop ventilation mode. Based on peripheral oxygen saturation and end-tidal carbon dioxide concentration measurements, it automatically adjusts minute ventilation, the fraction of inspired oxygen (FiO_2_) and the positive end-expiratory pressure (PEEP) breath-by-breath.^11^ INTELLiVENT®-ASV has been shown to safely ventilate patients in a variety of pathologies and to maintain ventilation settings and lung mechanics within the limits of protective mechanical ventilation, while reducing the necessity for medical and nursing adjustment.^12–14^

The objective of the present study was to assess the performance, in terms of lung-protective ventilation, of a closed-loop ventilation mode as compared to conventional mechanical ventilation in the resource-constrained setting of the COVID-19 pandemic.

## Methods

This prospective study was performed between March and May 2020 at the Institute of Intensive Care Medicine of the University Hospital Zurich, an academic tertiary care referral center. The study was approved by the cantonal ethics committee of Zurich (BASEC: 2020-01681) and informed consent was obtained from the patients or from their next of kin. The study complies with the Declaration of Helsinki, the Guidelines on Good Clinical Practice (GCP-Directive) issued by the European Medicines Agency as well as with Swiss law and regulatory authority requirements.

### Population

Patients were prospectively included in this study if they presented with (I) a SARS-CoV-2 infection that was laboratory confirmed by nucleic acid amplification according to the WHO-issued testing guidelines,^15^ and (II) a critical manifestation of COVID-19 requiring admission to an intensive care unit and treatment with invasive mechanical ventilation due to profound hypoxemia, complying with the Berlin definition for ARDS.^16^

### Study Design, Blinding and Ventilator Allocation

In the setting of the COVID-19 pandemic, resources at the Institute of Intensive Care Medicine had to be expanded to allow for care of a higher number of invasively ventilated COVID-19 patients. Therefore, in addition to the standard Hamilton-S1® (Hamilton Medical AG, Switzerland) mechanical ventilator, Draeger Evita® Infinity® V500 machines (Draegerwerk AG, Germany) had to be employed to mechanically ventilate COVID-19 patients. The standard mode of ventilation employed on the Hamilton-S1® ventilator was INTELLiVENT®-ASV 1.1 (closed-loop ventilation), and Biphasic Positive Airway Pressure Ventilation on the Draeger Evita® Infinity® V500 (conventional ventilation). Weaning from the Hamilton ventilator was approached via the INTELLiVENT®-ASV mode. For the Draeger ventilator, Biphasic Positive Airway Pressure and Spontaneous Continuous Positive Airway Pressure ventilation could be used. Ventilation times on other ventilation modes, during patient transport and during interventions, were disregarded in the final analysis to prevent potential biases. Physician and nursing staff in charge of treatment and care in the ICU were the same for both types of mechanical ventilator; no differentiation or splitting of teams dependent on ventilator expertise was undertaken. Medical and nursing staff were familiar with the use of both devices and ventilation modes as part of their daily routine, and had received an intensified refresher course on mechanical ventilation focusing on lung-protective ARDS ventilation in both closed-loop ventilation and conventional ventilation modes at the beginning of the pandemic. Institutional standard procedures were the same for both closed-loop and conventional mechanical ventilation, including but not limited to the proning of patients with a partial pressure of arterial oxygen (paO_2_) over FiO_2_ ratio (P/F ratio) <200 mmHg, the use of neuromuscular blocking agents in patients with a P/F ratio <150 mmHg or presenting an uncontrollable respiratory drive and vigorous breathing efforts under deep sedation, as well as the use of an esophageal pressure probe in patients at the limit of lung-protective ventilation or with a clinically assessed abnormal chest-wall compliance.

Patients were allocated, at the time-point of ICU admission, to either type of mechanical ventilator based on the availability of the latter by coordinating staff unaware of this study, without any further judgment or knowledge of the patients’ condition influencing this decision. Medical and nursing staff, including consulting physicians, were fully blinded to the existence of the present study. Further, the study team was blinded to the initial respirator allocation and had no influence on the decision.

### Data Collection and Lung-Protective Mechanical Ventilation Definition

All mechanical ventilators were attached to the patient data management system (MetaVision, iMDsoft, Israel) enabling a prospective, minute-by-minute collection of all mechanical ventilator settings and measurements. Changes in ventilator settings (respiratory rate, FiO_2_, PEEP, inspiratory pressure, support pressure, target shifts, PASV limit and INTELLiVENT®-ASV controllers) were algorithmically assessed on a minute-by-minute basis; thus, non-equal, temporally concomitant settings were noted as a change. To assure optimal ventilation and oxygenation of all patients, at least 1 blood gas analysis per 6 hours ICU was performed. Static driving pressure was measured at the time-point of endotracheal intubation as the difference of an inspiratory-and an expiratory-hold maneuver, subsequently ÷ static compliance was calculated as tidal volume static driving pressure. However, in order to enable a continuous, minute-by-minute assessment of driving pressure and mechanical power, we chose to employ their dynamic approximations in analogy to the previously published study by Urner et al.^17^ Peak inspiratory pressure was thus employed as a surrogate for plateau pressure. Consequently dynamic driving pressure was calculated as peak inspiratory pressure − positive end expiratory pressure, dynamic compliance as tidal volume ÷ dynamic driving pressure and dynamic mechanical power as 0.098 × respiratory rate × tidal volume × (peak inspiratory pressure − (0.5 × dynamic driving pressure)).

Lung-protective mechanical ventilation was defined and institutionally targeted as: a maximal tidal volume of 6-8 ml per kg of ideal body weight.^18,19^ a driving pressure <15 cmH_2_O,^20^ a plateau pressure <30 cmH_2_O,^21^ a paO_2_ ≥7.33 kPa or a peripheral oxygen saturation (SpO_2_) ≥88%^22,23^ under permissive hypercapnic ventilation with a lower pH limit of 7.25.^24^ Further, a mechanical power <17 J/min was defined as lung-protective for the setting of this study.^17,25^

### Statistical Analysis

Due to the breakthrough nature of this cohort study during the ongoing health crisis, no power calculations were undertaken. Comparisons of population characteristics were performed using the Wilcoxon Signed Rank and chi-squared test, as appropriate. A 2-sided *P* < 0.05 was considered statistically significant. For longitudinal analysis of mechanical ventilator parameters, lung mechanics and blood gas analyses, differences between time points and ventilation modes were tested using linear mixed effects model analysis. As independent variable fixed effects, time point and ventilation mode were entered into the model, respectively, with and without interaction terms, which were retained only if they were found to contribute to the model. As random effects, intercepts for subjects as well as per-subject random slopes for the effect on dependent variables were employed. *P* values were calculated using a likelihood ratio test of the full model, with the effect in question, against a “null model,” without the effect in question. *P* values for individual fixed effects were obtained by Satterthwaite approximation in a multi-dimensional model comprising time point and outcome status. Statistical analysis was performed via a fully scripted data management pathway using the R environment for statistical computing version 3.6.1.^26^ Values are given as medians with interquartile ranges or counts and percentages as appropriate.

## Results

### Demographics

**Figure 1.**
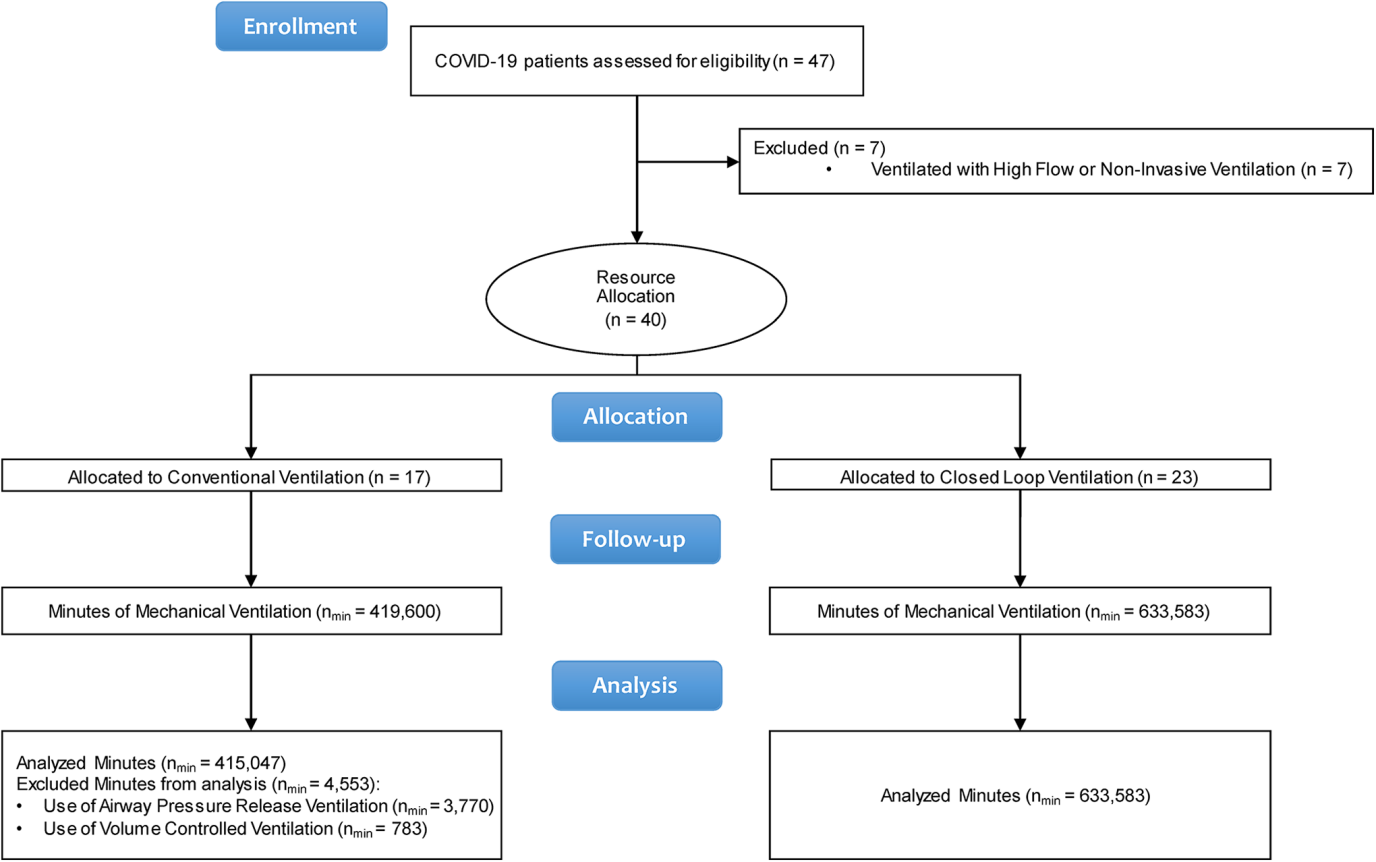
Study flow diagram.

Forty-seven patients with CARDS were admitted to the intensive care unit during the study period. Of these, 40 patients required invasive mechanical ventilation, were included in the study and allocated by study-blinded coordinating staff to either conventional ventilation (ConV) in 17 cases or closed-loop ventilation (CLoop) in 23 cases, as illustrated in Figure 1. At admission, patients were characterized by a P/F ratio of 92 [72-147] mmHg and a static compliance of 18 [11-25] cmH_2_O/ L, as presented in Table 1. Baseline characteristics were comparable for the 2 groups (Table 1). Overall time on mechanical ventilation was 11 [4-25] days and 28-day mortality amounted to 20% (Table 1). Only 1 patient died due to refractory respiratory failure, the leading causes of death were coagulopathy associated, with 4 patients deceasing due to intestinal ischemia and 1 due to central pulmonary embolism with right heart failure (Supplemental Table e1).

**Table 1.** Demographics, Baseline Characteristics at Intensive Care Unit Admission and Outcomes.^a^

	Overall population, N = 40	Conventional ventilation, N = 17	Closed loop ventilation, N = 23	*P*
Demographics				
Age [years]	61 [54-70]	59 [56-66]	66 [54-72]	0.331
Sex [male]	33 (83)	15 (88)	17 (78)	0.689
BMI [kg m^-2^]	28 [26-31]	29 [27-31]	27 [25-29]	0.151
SAPS II	36 [27-47]	36 [30-45]	36 [27-47]	0.837
SOFA	9 [6-10]	8 [6-10]	9 [6-10]	0.901
PaO_2_/ FiO_2_ [mmHg]	92 [72-147]	86 [66-137]	99 [77-147]	0.639
Time from first Symptoms to ICU Admission [days]	9 [5-14]	9 [6-15]	8 [5-10]	0.702
Time to ICU Admission from Hospital Admission [days]	0 [0-3]	1 [0-3]	0 [0-1]	0.126
Need for Vasopressors	19 (48)	8 (47)	11 (48)	0.899
Comorbidities				
Ischemic Heart Disease	8 (20)	1 (6)	7 (30)	0.347
Arterial Hypertension	23 (58)	9 (53)	14 (61)	0.859
Diabetes Mellitus	17 (43)	6 (35)	11 (48)	0.639
Chronic Pulmonary Disease	7 (18)	4 (24)	3 (13)	0.659
Chronic Renal Insufficiency	11 (28)	4 (24)	7 (30)	0.9
Solid Tumor	4 (10)	3 (18)	1 (4)	0.394
Immunosuppression^b^	7 (18)	4 (24)	3 (13)	0.659
Laboratory Parameters				
White Blood Cell Count [G/l]	8.2 [6.3-12.0]	7.3 [5.2-10.0]	10.0 [6.4-13.4]	0.123
Interleukin-6 [ng/l]	136 [90-507]	135 [73-796]	144.5 [91-238]	0.629
CRP [mg/l]	192 [102-283]	124 [62-245]	232 [165-287]	0.089
Creatinine [μmol/l]	91 [72-144]	91 [80-180]	91 [70-128]	0.941
D-Dimer [mg/l]	2725 [1020-4178]	1190 [720-3048]	3195 [1758-5668]	0.088
Arterial Blood Gas Analysis				
pH	7.28 [7.24-7.33]	7.30 [7.25-7.36]	7.28 [7.22-7.31]	0.172
paO_2_ [kPa]	8.3 [7.8-9.1]	8.3 [7.9-8.9]	8.3 [7.8-9.2]	0.825
paCO_2_ [kPa]	6.8 [6.0-7.5]	6.8 [6.0-7.7]	6.8 [6.2-7.4]	0.847
HCO_3_ [mmol/l]	23 [22-24]	23 [23-24]	24 [22-25]	0.988
Lactate [mmol/l]	0.9 [0.7-1.2]	0.9 [0.7-1.1]	0.9 [0.7-1.2]	1
Mechanical Ventilation Parameters				
FiO_2_ [%]	98 [95-100]	100 [96-100]	97 [95-100]	0.193
PEEP [cmH_2_O]	15 [14-17]	15 [11-16]	16 [14-17]	0.245
Tidal Volume/ IBW [ml/kg]	7.5 [6.5-8.8]	6.6 [6.5-8.1]	8.0 [6.8-9.0]	0.065
Static Driving Pressure [cmH_2_O]	17 [15-20]	21 [17-24]	16 [14-18]	0.001
Respiratory Rate [1/min]	28 [23-32]	30 [27-34]	27 [22-32]	0.079
Minute Volume Ventilation [l/min]	9.6 [8.4-11.0]	11.2 [8.6-13.8]	9.5 [8.3-10.4]	0.095
Peak Inspiratory Pressure [cmH_2_O]	29 [26-33]	33 [30-36]	27 [25-31]	0.005
Static Mechanical Power [J/min]	21 [19-24]	24 [21-31]	19 [16-22]	0.004
Static Compliance [ml/cmH_2_O]	18 [11-25]	16 [11-20]	21 [12-26]	0.203
Outcome				
Duration of Mechanical Ventilation [days]	11 [4-25]	12 [6-24]	10 [4-23]	0.837
Length of ICU stay [days]	18 [8-32]	19 [7-32]	18 [9-33]	0.547
28-Day Mortality	8 (20)	2 (12)	6 (26)	0.472

Abbreviations: BMI, Body Mass index; SAPS, Simplified Acute Physiology Score; SOFA, Sequential Organ Failure Assessment; CRP, C-reactive protein; PCT, Procalcitonin; LDH, Lactate dehydrogenase; HCO_3_, Bicarbonate; paO_2_, partial pressure of arterial oxygen; paCO_2_, partial pressure of arterial carbon dioxide; FiO_2_, Fraction of inspired oxygen; PEEP, Positive End-Expiratory Pressure; IBW, Ideal Body Weight; ICU, Intensive Care Unit.

^a^ Quantitative data are expressed as median [interquartile range] or counts (and percentages) as appropriate.

^b^ Immunosuppression was defined as any of the following: Hematological Malignancy, Human Immunodeficiency Virus, Hepatitis B or C, Prescribed Immunosuppressive Medication.

### Overall Mechanical Ventilation

Overall, 1,048,630 minutes or 728 days of cumulative mechanical ventilator time were analyzed (Table 2). Patients in the CLoop group were ventilated with slightly higher tidal volumes normalized to the ideal body weight (IBW), leading to lower partial pressures of arterial carbon dioxide (paCO_2_) at a clinically comparable pH as opposed to the ConV group (Table 2). Regarding oxygenation, the CLoop group presented overall lower paO_2_ levels at lower PEEP and FiO_2_ settings than did the ConV group, but was nevertheless characterized by a higher P/F ratio with 199 [152-251] mmHg versus 168 [126-216] mmHg in the ConV group (*P* < 0.001) (Table 2).

**Table 2.** Arterial Blood Gas Analyses and Mechanical Ventilator Parameters Over the Course of Mechanical Ventilation.^a^

	Overall population, N = 40	Conventional ventilation, N = 17	Closed loop ventilation, N = 23	*P*
Initial 7 Days of Mechanical Ventilation				
n [minutes]	348319	173626	174693	
pH	7.37 [7.31-7.41]	7.37 [7.31-7.42]	7.36 [7.31-7.40]	<0.001
paO_2_ [kPa]	9.5 [8.8-10.5]	9.8 [8.8-10.9]	9.3 [8.8-10.2]	<0.001
SpO_2_ [%]	93 [91-95]	94 [92-96]	93 [91-94]	<0.001
paCO_2_ [kPa]	5.9 [5.3-6.8]	5.9 [5.2-6.8]	6.0 [5.3-6.8]	0.954
PaO_2_/ FiO_2_ Ratio [mmHg]	165 [121-209]	161 [115-207]	169 [127-210]	0.005
FiO_2_ [%]	43 [35-55]	45 [35-57]	42 [33-54]	<0.001
PEEP [cmH_2_O]	11 [9-14]	11 [9-14]	11 [9-14]	<0.001
Tidal Volume [ml]	385 [304-446]	349 [252-429]	394 [321-450]	<0.001
Tidal Volume/ IBW [ml/kg]	5.7 [5.1-6.5]	5.7 [4.8-6.6]	5.8 [5.3-6.5]	<0.001
Respiratory Rate [1/min]	23 [18-26]	24 [20-27]	21 [18-25]	<0.001
Minute Ventilation [l/min]	8.6 [6.8-10.5]	9.5 [7.3-11.2]	7.9 [6.8-9.5]	<0.001
Peak Inspiratory Pressure [cmH_2_O]	25 [21-28]	27 [23-30]	23 [20-26]	<0.001
Dynamic Driving Pressure [cmH_2_O]	13 [10-16]	15 [12-19]	12 [10-14]	<0.001
A-a Gradient [mmHg]	184 [125-286]	200 [137-302]	172 [117-266]	<0.001
Alveolar Dead Space [ml]	41 [19-74]	45 [22-78]	40 [18-70]	0.002
Dynamic Compliance [ml/ cmH_2_O]	29. [22-40]	25 [20-35]	33 [24-43]	<0.001
Dynamic Mechanical Power [J/min]	14 [11-19]	17 [10-23]	13 [11-16]	<0.001
Overall Time on Mechanical Ventilation				
n [minutes]	1048630	415047	633583	
pH	7.38 [7.33-7.43]	7.38 [7.32-7.43]	7.38 [7.33-7.42]	<0.001
paO_2_ [kPa]	9.8 [9.0-11.0]	9.9 [9.0-11.1]	9.7 [9.0-10.9]	0.002
SpO_2_ [%]	94 [92-96]	94 [92-96]	94 [92-96]	<0.001
paCO_2_ [kPa]	6.0 [5.1-7.0]	6.0 [5.1-7.2]	6.0 [5.0-6.9]	<0.001
PaO_2_/ FiO_2_ Ratio [mmHg]	185 [141-241]	168 [126-216]	199 [152-251]	<0.001
FiO_2_ [%]	38 [30-50]	42 [34-54]	36 [30-47]	<0.001
PEEP [cmH_2_O]	9 [6-12]	9 [7-12]	9 [6-11]	<0.001
Tidal Volume [ml]	394 [304-482]	355 [278-450]	406 [317-492]	<0.001
Tidal Volume/ IBW [ml/kg]	5.8 [4.9-6.8]	5.8 [4.7-6.9]	5.8 [4.9-6.7]	<0.001
Respiratory Rate [1/min]	25 [20-28]	25 [20-28]	24 [20-29]	0.118
Minute Ventilation [l/min]	9.6 [7.4-11.9]	9.9 [7.5-12.0]	9.3 [7.4-11.9]	<0.001
Peak Inspiratory Pressure [cmH_2_O]	24 [19-28]	26 [20-29]	23 [19-27]	<0.001
Dynamic Driving Pressure [cmH_2_O]	14 [10-18]	15 [12-19]	13 [10-17]	<0.001
A-a Gradient [mmHg]	153 [98-234]	184 [123-278]	134 [90-204]	<0.001
Alveolar Dead Space [ml]	42 [13-76]	47 [15-82]	40 [12-74]	<0.001
Dynamic Compliance [ml/ cmH_2_O]	27 [19-42]	25 [19-38]	29 [20-44]	<0.001
Dynamic Mechanical Power [J/min]	15 [10-20]	16 [9-22]	14 [10-19]	<0.001

Abbreviations: paO_2_—partial pressure of arterial oxygen; SpO_2_, Peripheral oxygen saturation; paCO_2_, Partial pressure of arterial carbon dioxide; FiO_2_, Fraction of inspired oxygen; PEEP, Positive End-Expiratory Pressure; IBW, Ideal Body Weight; A-a Gradient, Alveolar-arterial Gradient.

^a^ Quantitative data are expressed as median [interquartile range].

Further, and as shown in Table 2, peak inspiratory pressure, dynamic driving pressure as well as dynamic mechanical power could be held systematically lower in the CLoop than in the ConV group (*P* < 0.001). This was accompanied by a decreased alveolar dead space and alveolo-arterial gradient in the CLoop group (*P* < 0.001) (Table 2).

### Initial CARDS Mechanical Ventilation

**Figure 2. (A to F):**
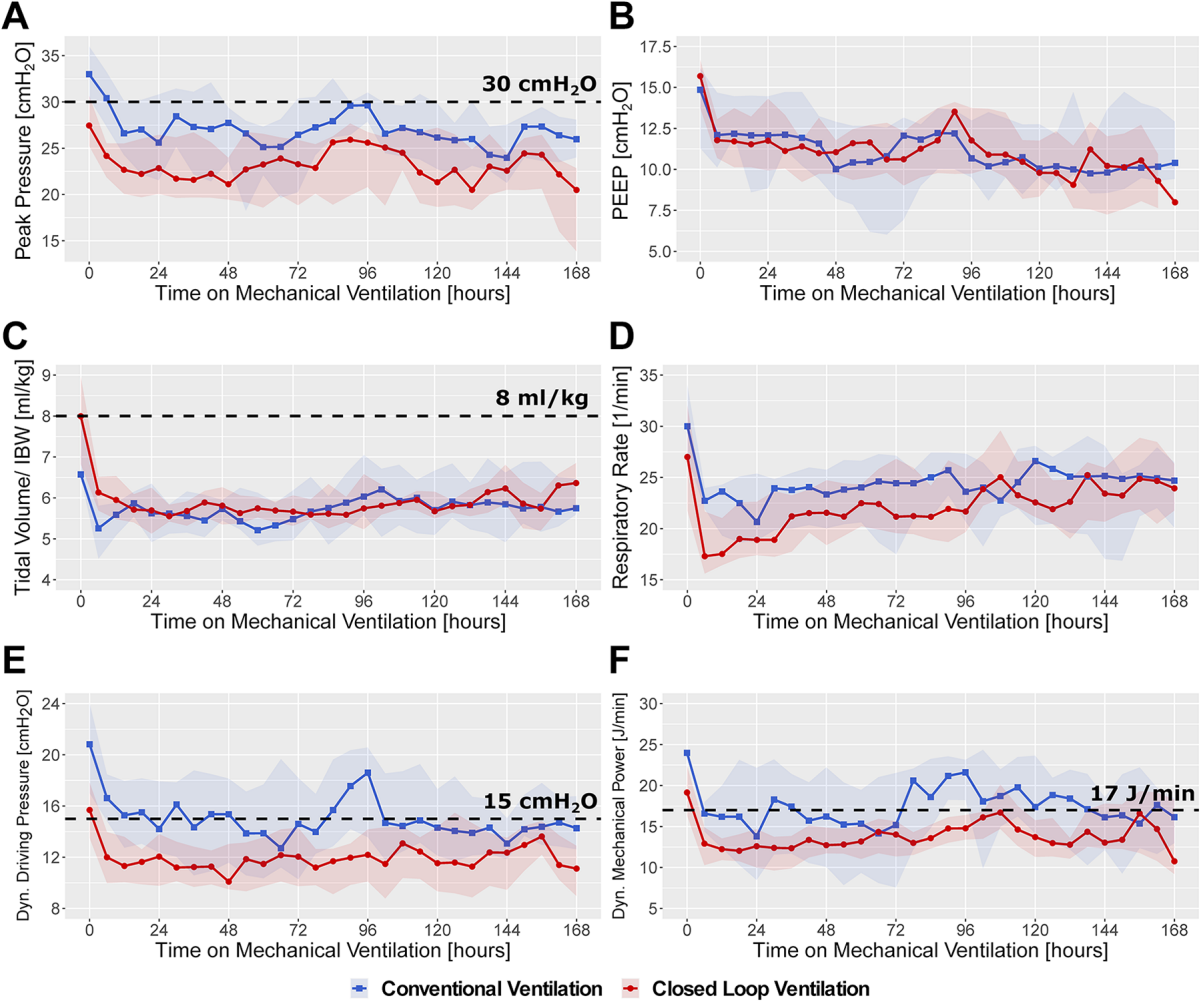
Main ventilatory characteristics of COVID-19 ARDS patients ventilated with conventional ventilation mode versus closed loop ventilation over the first week of ventilation. For ease of visualization, individual patient data were averaged into 6-hour intervals. Lines represent median values, shaded areas the interquartile ranges.

During the initial 7 days post intubation, patients in the CLoop group experienced systematically lower peak inspiratory pressures (*P* < 0.001), respiratory rate (*P* < 0.001), dynamic driving pressure (*P* < 0.001) and dynamic mechanical power (*P* < 0.001), while achieving higher dynamic compliance (*P* < 0.01) and lower alveolar dead space (*P* < 0.002) than did patients in the ConV group, as evidenced in Figure 2, Table 2, Supplemental Figures e1, e2 and Supplemental Tables e2, e3, e4.

### Lung-Protective Ventilation

**Figure 3.**
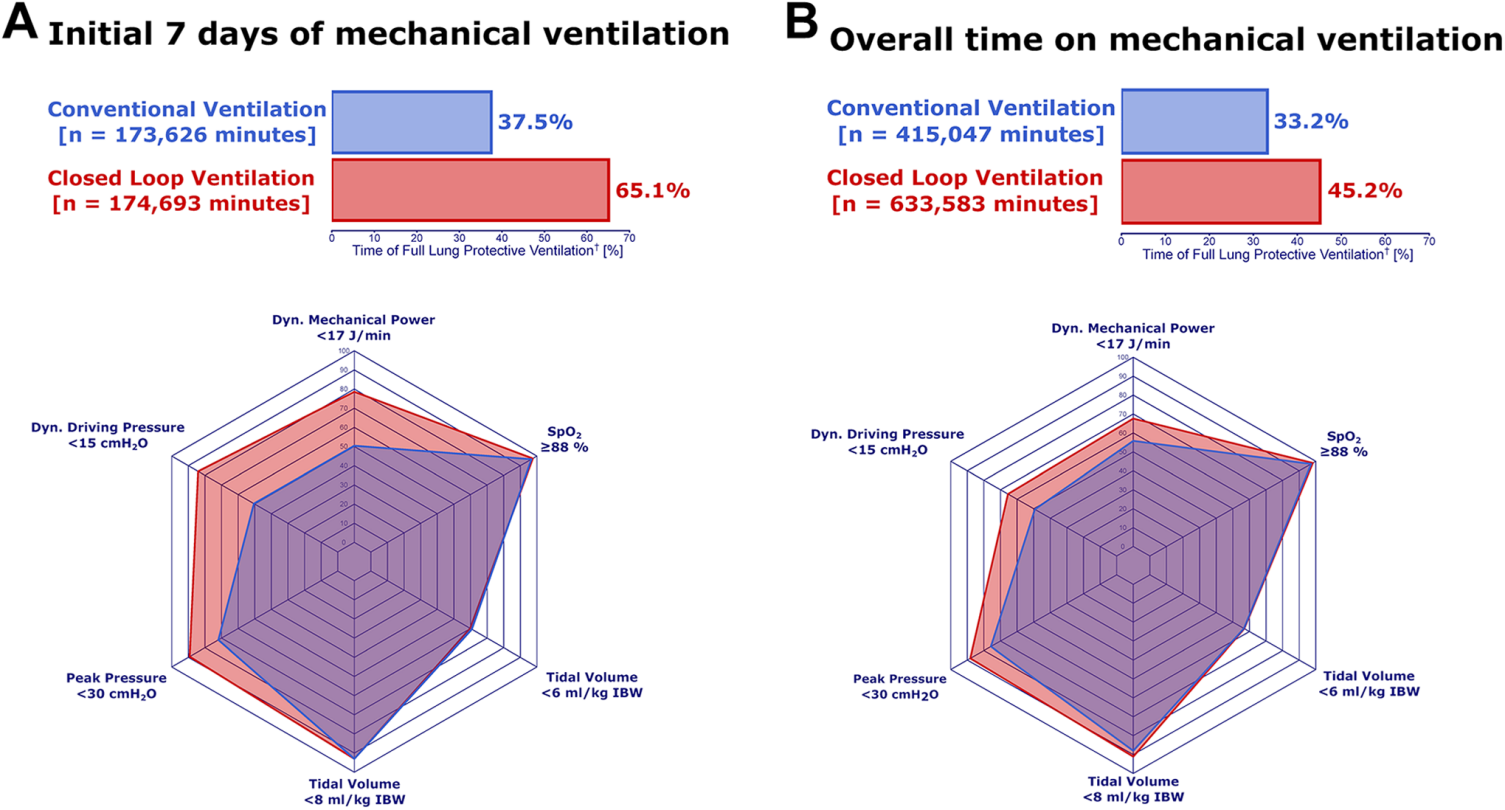
Bar plots and spider diagrams assessing the percentage of time of lung-protective mechanical ventilation (A) in reference to the overall mechanical ventilation time (B) in COVID-19 ARDS patients ventilated with conventional and closed loop ventilation. ^†^Full lung protective ventilation was defined as the conjoined target of tidal volumes <8 ml/ kg, peak inspiratory pressure <30 cmH_2_O, dynamic driving pressure <15 cmH_2_O, peripheral oxygen saturation >88% and a dynamic mechanical power <17 J/min.

The dynamic driving pressure in the CLoop group was maintained at <15 cmH_2_O for 84% of the time during the initial week, and 66% for the overall ventilation time, as opposed to 51% (*P* < 0.001) and 49% (*P* < 0.001), respectively, for dynamic driving pressures in the ConV group (Figure 3, Supplemental Tables e5, e6). Further, dynamic mechanical power could be held to <17 J/min for 79% of the ventilation time during the initial week in the CLoop group, compared to 50% achieved in the ConV group (*P* < 0.001); this difference was also patent when the overall ventilation time between groups was compared (*P* < 0.001). Additionally, the percentage of time with a peak inspiratory pressure <30 cmH_2_O (*P* < 0.001) was also higher in the CLoop group. Simultaneously, paO_2_ levels remained ≥7.33 kPa for 99% and the SpO_2_ ≥88% for 99% of the ventilation time in the CLoop group versus 99% (*P* < 0.001) and 97% (*P* < 0.001) in the ConV group, respectively. Finally, patients in the CLoop group were successfully ventilated in a fully protective fashion, with conjoint tidal volumes <8 ml/kg IBW, dynamic driving pressures <15 cmH_2_O, peak inspiratory pressures <30 cmH_2_0, SpO_2_ ≥88% and dynamic mechanical power <17 J/min, over 45% of the time, 63% during the first week, as opposed to 33% (OR 1.79; 95% CI 1.76-1.82; *P* < 0.001) and 38% (OR 1.22; 95% CI 1.21-1.23; *P* < 0.001), respectively, in the ConV group.

### Changes in Settings

In patients ventilated in the ConV group 7 [3-12] changes per day had to be manually implemented to adapt mechanical ventilator settings as opposed to 4 [2-7] in the CLoop group (*P* = 0.02). In comparison, the automated algorithm in the CLoop group adapted the ventilator settings every 2.8 [2.3-3.6] minutes.

## Discussion

In this prospective study, closed-loop mechanical ventilation (CLoop) was compared with conventional mechanical ventilation (ConV) in COVID-19 ARDS (CARDS) on a minute-by-minute basis. The CLoop group was fully protectively ventilated concerning tidal volume, driving pressure, peak inspiratory pressure, peripheral oxygen saturation and mechanical power, for 65% of the first week and 45% of their overall ventilation time, as opposed to 38% and 33%, respectively, for those in the ConV group. This was achieved with a concomitant decrease in the need for manual adjustment of the ventilator settings in the CLoop compared to the ConV group.

Protective invasive mechanical ventilation has become a clear goal in ARDS therapy in order to limit strain to the “baby lung” and prevent VILI.^3^ In this setting, reduced tidal volume ventilation patterns with 4 to 8 ml/kg IBW tidal volumes have become standard of care.^18,19^ Nonetheless, the implementation of these standards is still not universal; in the recent LUNG SAFE study only two-thirds of the ARDS patients received tidal volumes below 8 ml/kg IBW^27^ and recent data show only 23% of patients with CARDS being ventilated with tidal volumes below 6 ml/kg IBW.^28^ The present study reports a limitation of tidal volumes below 8 kg/ml IBW for 90% and below 6 ml/kg for 57% of the mechanical ventilation time in the ConV group, reflecting the excellent training and understanding of protective ventilation among ICU staff. Nonetheless, and regardless of the evident proficiency of the medical and nursing staff, CLoop was superior to ConV concerning settings for inspiratory pressure and dynamic driving pressure. These 2 variables act as surrogate parameters for transpulmonary pressure and pulmonary distension, and their limitation at 30 cmH_2_O^21^ and 15 cmH_2_O,^17,20^ respectively, has been correlated with systematic mortality reductions in ARDS. Further, recent research has emphasized the importance of elevated cycling frequencies as amplifiers of the static strain induced by tidal volumes and driving pressures leading to the stress failure of stress-bearing alveolar micro-elements.^3,29^ The observed reduced respiratory rate in the CLoop group during the acute phase of CARDS ventilation, as opposed to the higher respiratory frequencies in the ConV group, could thus be a further protective factor. Interestingly, and in contrast to ConV, CLoop enabled normoxic ventilation at higher P/F ratios while reducing FiO_2_ levels. Elevated FiO_2_ settings have been previously postulated as an exacerbating variable to alveolar barrier dysfunction, aggravating the degree of VILI, while their limitation has shown protective effects.^30–33^

The concept of mechanical power is a relatively new and holistic approach to quantifying the energetic strain delivered to the lung during ventilation.^34,35^ Mechanical power combines the effects of all main ventilatory variables, and powers above 17 J/min have been associated with worse outcomes in ARDS.^17,25^ Even though mechanical power calculation is not included in the CLoop algorithm, this type of ventilation mode managed to limit mechanical power to under 17 J/min for 79% of ventilation time while ConV did so for only 50% of the time, a value similar to the mechanical power reported in large ARDS cohorts.^17^ Interestingly, the percentage of time under lung-protective mechanical ventilation in the CLoop group, especially regarding mechanical power, was reduced during the first week in comparison to the overall time, probably indicating the difficulty of the closed-loop algorithm to counterbalance spontaneous ventilation.

Integrating lung-protective ventilation approaches into routine mechanical ventilation is a complex and resource-intensive endeavor, even for experienced clinicians and nurses.^27,36^ The pandemic triggered by SARS-CoV-2 has overwhelmed hospitals, leading to a lack of personnel and resources in ICUs.^5–7^ Mortalities in mechanically ventilated CARDS patients have been reported to oscillate between 20%-80% in this setting,^1,8,28,37,38^ with most lying substantially above the 30% reported in classic ARDS.^10^ Reasons for the variability of mortality are manifold, probably reflecting the heterogeneity of treatment strategies, especially regarding off-label therapies, patient triage before ICU, variable degrees of resources and staffing limitation as well as ventilation strategies, including ventilator sharing, among others.^1,8,28,37–39^ Nonetheless, the elevated incidence of barotraumas in CARDS as compared to classical ARDS may mainly be rooted in the exhaustion of personal resources, coupled with the recruitment of staff who are inexperienced in ARDS treatment.^8,9,40^ The thorough implementation of lung-protective protocols could explain the exceptionally low 28-day mortality of only 20% in this cohort. Most notably no ventilator associated barotraumas were observed in this cohort and only 1 patient died of refractory respiratory failure.

In analogy to previous studies on CLoop, the number of manual changes in mechanical ventilator settings by caregivers was lower with CLoop than with ConV.^12^ This added efficiency could reduce strain on nursing and medical staff, especially within the framework of the COVID-19 pandemic. Further, it allows inexperienced ICU staff to implement lung-protective ventilation strategies without the need for exhaustive training and considerations of the high heterogeneity of CARDS.^2,41^ Experienced caregivers are rare and the implementation of their know-how is resource-intensive.^36^

The present study has to acknowledge certain limitations. First, this study was not a classic and truly randomized controlled trial; nevertheless, the study design chosen was devised to maximally reduce biases. Second, the primary end-point of the study, albeit of pathophysiological relevance, does not prove a clinical benefit of CLoop over ConV. Nonetheless, this study describes continuous, minute-by-minute sampled ventilatory data, something unaccomplished before over such a long period of mechanical ventilation in ARDS. This, in turn, allows a faithful representation of both ventilation strategies over time, as opposed to the reporting of daily sampled ventilation data, and counterbalances the relatively low number of recruited patients. Third, the relevance of the reduction in ventilator interactions described was not directly correlated to the bedside experience of the ICU staff; as such, the degree of reduction in effective strain on medical and nursing personnel is not quantifiable. Fourth, it can be argued, that the present study only regarded CLoop comparing it with pressure-controlled ventilation, and disregarding the still widely used volume-controlled ventilation. However, there is no clear evidence favoring any mode over pressure control in ARDS.^42,43^ Fifth, the use of peak pressure as a surrogate parameter for plateau pressure may be a relevant confounder for the interpretability of the data. Nevertheless, and as previously shown, dynamic driving pressure and mechanical power have a clinical repercussion on outcome similar to static driving pressure and mechanical power.^17^ Further, there is no current method for faithfully assessing static plateau pressure in a continuous approach. Sixth, it could be argued that ICU staff were not accustomed to pressure-controlled ventilation and lung-protective strategies, leading to the observed inferiority of ConV. However, the implementation of mechanical ventilation refreshers for the ICU staff previous to this study’s initiation as well as the high degree of implementation of lung-protective strategies in the ConV group compared to what is mentioned in the literature, argue against this point. Finally, while the implementation and acceptance of closed-loop ventilation is widespread in the ICU setting in which this study was performed, the *de-novo* implementation of this tool in other ICUs may face resistance due to its novelty and an intrinsic reluctance to use automatic tools in ICU settings. As shown here, the safety and efficacy of closed-loop ventilation as well as the reduction of workload should be strong enough arguments to support a supervised implementation of this technology, especially in the setting of a pandemic.

## Conclusion

In conclusion, closed-loop ventilation, when compared to conventional mechanical ventilation, is associated with a higher degree of lung-protective ventilation, coupled with less hypoxemic time, while reducing the number of mechanical ventilator setting adjustments necessary in CARDS during an early highpoint of the COVID-19 pandemic.

## Supplemental Material

Supplemental Material, sj-pdf-1-jic-10.1177_08850666211024139 - Closed-Loop Versus Conventional Mechanical Ventilation in COVID-19 ARDS Click here for additional data file. Supplemental Material, sj-pdf-1-jic-10.1177_08850666211024139 for Closed-Loop Versus Conventional Mechanical Ventilation in COVID-19 ARDS by Pedro David Wendel Garcia, Daniel Andrea Hofmaenner, Silvio D. Brugger, Claudio T. Acevedo, Jan Bartussek, Giovanni Camen, Patrick Raphael Bader, Gregor Bruellmann, Johannes Kattner, Christoph Ganter, Reto Andreas Schuepbach and Philipp Karl Buehler in Journal of Intensive Care Medicine
