# Supplementary material for: Closed-Loop Versus Conventional Mechanical Ventilation in COVID-19 ARDS
Source: J Intensive Care Med. 2021 Oct;36(10):1184–93. doi: 10.1177/08850666211024139 (PMC8442133; doi:10.1177/08850666211024139)
Supplement: Supplemental Material, sj-pdf-1-jic-10.1177_08850666211024139 - Closed-Loop Versus Conventional Mechanical Ventilation in COVID-19 ARDS [file sj-pdf-1-jic-10.1177_08850666211024139.pdf]

Supplemental Digital Content

Closed-loop versus conventional mechanical ventilation in COVID-19 ARDS

Pedro David Wendel Garcia MSc, Daniel Andrea Hofmaenner MD, Silvio D. Brugger MD PhD, Claudio T. Acevedo MD, Jan Bartussek PhD, Giovanni Camen MD, Patrick Raphael Bader MD, Gregor Bruellmann MD, Johannes Kattner MD, Christoph Ganter MD, Reto Andreas Schuepbach MD, Karl Philipp Buehler MD

- **Supplemental Table e1:** Causes of death and incidence of barotraumas
- **Supplemental Table e2:** Mixed Effect Model of the main ventilatory characteristics stratified by ventilation mode over the first week of ventilation.
- **Supplemental Figure e1:** Oxygenation parameters stratified by ventilation mode over the first week of ventilation.
- **Supplemental Table e3:** Mixed Effect Model of oxygenation parameters stratified by ventilation mode over the first week of ventilation.
- **Supplemental Figure e2:** Ventilation parameters stratified by ventilation mode over the first week of ventilation.
- **Supplemental Table e4:** Mixed Effect Model of ventilation parameters stratified by ventilation mode over the first week of ventilation.
- **Supplemental Table e5:** Percentage of time of lung-protective mechanical ventilation during the first 7 days of mechanical ventilation
- **Supplemental Table e6:** Percentage of time of lung-protective mechanical ventilation during the overall time of mechanical ventilation

Supplemental Table e1: Causes of death and incidence of barotraumas

	Overall Population N =40	Conventional Ventilation N = 17	Closed Loop Ventilation N = 23
**Causes of death**	8 (20%)	2 (12%)	6 (26%)
**Refractory respiratory failure**	1 (3%)	1 (6%)	0 (0%)
**Refractory septic shock**	2 (5%)	1 (6%)	1 (4%)
**Intestinal ischaemia**	4 (10%)	0 (0%)	4 (17%)
**Pulmonary embolism with right heart failure**	1 (3%)	0 (0%)	1 (4%)
**Incidence of Barotraumas**	0 (0%)	0 (0%)	0 (0%)

Supplemental Table e2: Mixed Effect Model of the main ventilatory characteristics stratified by ventilation mode over the first week of ventilation.

	**0h - CV**	**0h - CLV**	**6h - CV**	**6h - CLV**	**12h - CV**	**12h - CLV**	**18h - CV**	**18h - CLV**	**24h - CV**	**24h - CLV**	**30h - CV**	**30h - CLV**	**36h - CV**	**36h - CLV**	**42h - CV**	**42h - CLV**	**48h - CV**	**48h - CLV**	**54h - CV**	**54h - CLV**
**Peak Pressure [cmH_2O]**	33 [30 - 36]	27 [25 - 31]	30 [27 - 33]	24 [22 - 26]	27 [24 - 30]	23 [20 - 26]	27 [22 - 30]	22 [20 - 26]	26 [22 - 31]	23 [20 - 26]	28 [25 - 31]	22 [20 - 26]	27 [22 - 31]	22 [20 - 27]	27 [21 - 32]	22 [20 - 26]	28 [24 - 29]	21 [19 – 24]	27 [18 - 28]	23 [19 - 27]
**PEEP [cmH_2O]**	15 [11 - 16]	16 [14 - 17]	12 [10 - 15]	12 [11 - 13]	12 [12 - 14]	12 [10 - 14]	12 [9 - 15]	12 [10 - 13]	12 [9 - 14]	12 [10 - 14]	12 [11 - 15]	11 [10 - 13]	12 [10 - 15]	11 [9 - 12]	12 [9 - 13]	11 [10 - 13]	10 [8 - 13]	11 [10 - 12]	10 [8 - 13]	12 [9 - 12]
**Tidal Volume/ IBW [ml/kg]**	6.6 [6.5 - 8.1]	8.0 [6.8 - 9.0]	5.26 [4.51 - 5.81]	6.13 [5.69 - 6.52]	5.59 [5.09 - 5.88]	5.95 [5.48 - 6.54]	5.87 [5.54 - 6.15]	5.71 [5.4 - 6.09]	5.62 [4.92 - 6.35]	5.69 [5.13 - 6.25]	5.61 [5.13 - 5.84]	5.55 [5.42 - 5.83]	5.56 [5.15 - 6.16]	5.68 [5.32 - 5.91]	5.45 [5.04 - 6.07]	5.89 [5.54 - 6.17]	5.73 [5.45 - 5.95]	5.81 [5.35 - 6.26]	5.43 [5.23 - 6.25]	5.63 [5.37 - 6.06]
**Respiratory Rate [1/min]**	30 [27 - 34]	27 [22 - 32]	23 [22 - 24]	17 [16 - 22]	24 [19 - 24]	18 [16 - 21]	22 [19 - 25]	19 [17 - 23]	21 [19 - 25]	19 [17 - 23]	24 [20 - 25]	19 [17 - 24]	24 [20 - 25]	21 [18 - 23]	24 [20 - 26]	22 [20 - 24]	23 [20 - 26]	22 [18 - 25]	24 [20 - 27]	21 [19 - 24]
**Dynamic Driving Pressure [cmH_2O]**	21 [17 - 24]	16 [14 - 18]	17 [15 - 18]	12 [10 - 14]	15 [13 - 18]	11 [10 - 14]	16 [13 - 18]	12 [10 - 13]	14 [12 - 18]	12 [10 - 14]	16 [12 - 18]	11 [10 - 13]	14 [11 - 19]	11 [10 - 13]	15 [11 - 18]	11 [10 - 13]	15 [12 - 18]	10 [10 - 13]	14 [11 - 17]	12 [9 - 13]
**Dynamic Mechanical Power [J/min]**	24 [21 - 31]	19 [16 - 22]	17 [14 - 18]	13 [10 - 15]	16 [12 - 20]	12 [11 - 14]	16 [10 - 23]	12 [11 - 14]	14 [8 - 22]	13 [10 - 15]	18 [12 - 23]	12 [10 - 14]	17 [12 - 21]	12 [10 - 14]	16 [11 - 20]	13 [11 - 15]	16 [10 - 22]	13 [12 - 15]	15 [8 - 20]	13 [12 - 15]

	60h - CV	60h - CLV	66h - CV	66h - CLV	72h - CV	72h - CLV	78h - CV	78h - CLV	84h - CV	84h - CLV	90h - CV	90h - CLV	96h - CV	96h - CLV	102h - CV	102h - CLV	108h - CV	108h - CLV	114h - CV	114h - CLV
**Peak Pressure [cmH_2O]**	25 [22 – 27]	23 [19 - 27]	25 [20 - 28]	24 [20 - 26]	26 [20 - 30]	23 [19 - 26]	27 [24 - 31]	23 [20 - 25]	28 [26 - 33]	26 [19 - 26]	30 [26 - 30]	26 [20 - 28]	30 [28 - 31]	26 [21 - 28]	27 [24 - 30]	25 [19 - 26]	27 [24 - 30]	25 [23 - 27]	27 [24 - 31]	22 [19 - 27]
**PEEP [cmH_2O]**	10 [6 - 13]	12 [10 - 13]	11 [6 - 13]	11 [10 - 13]	12 [7 - 13]	11 [9 - 12]	12 [9 - 13]	12 [10 - 13]	12 [10 - 14]	12 [10 - 14]	12 [10 - 14]	14 [10 - 14]	11 [10 - 13]	12 [10 - 14]	10 [8 - 13]	11 [10 - 14]	10 [10 - 13]	11 [10 - 13]	11 [8 - 13]	10 [8 - 12]
**Tidal Volume/ IBW [ml/kg]**	5.2 [4.8 - 6.1]	5.8 [5.5 - 6.0]	5.3 [4.9 - 5.9]	5.7 [5.4 - 5.9]	5.5 [5.0 - 6.2]	5.7 [5.3 - 6.1]	5.7 [5.4 - 6.5]	5.6 [5.5 – 6.0]	5.8 [4.9 - 6.0]	5.6 [5.3 - 6.3]	5.9 [5.2 - 6.6]	5.6 [5.4 - 6.0]	6.0 [5.6 - 7.1]	5.7 [5.2 - 6.6]	6.2 [5.1 - 6.7]	5.8 [5.6 - 6.3]	5.9 [5.3 - 6.8]	5.9 [5.7 – 6.0]	6.0 [5.1 - 6.4]	6.0 [5.7 - 6.2]
**Respiratory Rate [1/min]**	24 [21 - 27]	22 [20 - 24]	25 [19 - 27]	22 [20 - 26]	24 [18 - 27]	21 [20 - 27]	24 [21 - 26]	21 [19 - 26]	25 [21 - 27]	21 [19 - 24]	26 [23 - 27]	22 [19 - 27]	24 [21 - 27]	22 [20 - 26]	24 [18 - 26]	24 [22 - 27]	23 [21 - 25]	25 [23 - 30]	25 [22 - 28]	23 [22 - 27]
**Dynamic Driving Pressure [cmH_2O]**	14 [10 - 18]	11 [10 - 14]	13 [11 - 20]	12 [10 - 14]	15 [10 - 18]	12 [10 - 14]	14 [12 - 17]	11 [10 - 13]	16 [13 - 20]	12 [9 - 13]	18 [14 - 20]	12 [10 - 13]	19 [14 - 21]	12 [10 - 14]	15 [12 - 19]	11 [9 - 14]	14 [12 - 19]	13 [10 - 14]	15 [13 - 19]	12 [10 - 14]
**Dynamic Mechanical Power [J/min]**	15 [9 - 19]	13 [11 – 15]	14 [8 - 21]	14 [12 - 16]	15 [8 - 22]	14 [11 - 16]	21 [12 - 22]	13 [12 - 14]	19 [15 - 23]	14 [12 - 15]	21 [18 - 24]	15 [13 - 16]	22 [18 - 23]	15 [13 - 16]	18 [10 - 24]	16 [14 - 17]	19 [15 - 22]	17 [15 - 20]	20 [16 - 23]	15 [13 - 18]

	120h - CV	120h - CLV	126h - CV	126h - CLV	132h - CV	132h - CLV	138h - CV	138h - CLV	144h - CV	144h - CLV	150h - CV	150h - CLV	156h - CV	156h - CLV	162h - CV	162h - CLV	168h - CV	168h - CLV	p - Group	p - Time
**Peak Pressure [cmH_2O]**	26 [23 - 30]	21 [19 - 27]	26 [22 - 28]	23 [19 - 27]	26 [23 - 30]	21 [18 - 25]	24 [23 - 27]	23 [20 - 27]	24 [2 - 28]	23 [21 - 24]	27 [24 - 29]	24 [20 - 26]	27 [24 - 29]	24 [21 - 26]	26 [24 - 28]	22 [16 - 24]	26 [24 - 28]	20 [14 - 23]	< 0.0001	< 0.0001
**PEEP [cmH_2O]**	10 [10 - 13]	10 [8 - 12]	10 [9 - 12]	10 [10 - 10]	10 [10 - 15]	9 [8 - 11]	10 [9 - 12]	11 [8 - 13]	10 [9 - 14]	10 [7 - 12]	10 [9 - 15]	10 [8 - 12]	10 [9 - 14]	11 [8 - 13]	10 [9 - 14]	9 [8 - 11]	10 [9 - 13]	8 [5 - 11]	< 0.0001	< 0.0001
**Tidal Volume/ IBW [ml/kg]**	5.7 [5.1 - 6.7]	5.7 [5.5 - 6.2]	5.9 [5.5 - 6.8]	5.8 [5.6 – 6.0]	5.8 [5.2 - 6.4]	5.8 [5.6 - 6.2]	5.9 [5.1 – 7.0]	6.1 [5.6 - 6.4]	5.8 [4.9 - 6.6]	6.2 [5.9 - 6.8]	5.7 [5.4 - 6.9]	5.9 [5.3 - 6.4]	5.8 [5.2 - 6.9]	5.7 [5.2 - 6.1]	5.7 [5.3 - 6.5]	6.3 [5.7 - 6.6]	5.8 [5.6 - 6.5]	6.4 [5.7 - 6.9]	0.04	< 0.0001
**Respiratory Rate [1/min]**	27 [20 - 28]	23 [21 - 27]	26 [20 - 27]	22 [20 - 25]	25 [20 - 28]	23 [19 -25]	25 [19 - 29]	25 [21 - 26]	25 [17 - 28]	23 [21 - 26]	25 [17 - 25]	23 [21 - 28]	25 [21 - 26]	25 [22 - 29]	25 [22 - 28]	25 [20 - 28]	25 [20 - 26]	24 [22 - 26]	0.02	< 0.0001
**Dynamic Driving Pressure [cmH_2O]**	14 [12 - 17]	12 [9 - 14]	14 [12 - 18]	12 [9 - 15]	14 [12 - 17]	11 [9 - 15]	14 [12 - 17]	12 [10 - 14]	13 [12 - 15]	12 [9 - 14]	14 [14 - 17]	13 [11 - 14]	14 [13 - 18]	14 [12 - 15]	15 [13 - 17]	11 [10 - 14]	14 [13 - 17]	11 [9 - 13]	< 0.0001	0.09
**Dynamic Mechanical Power [J/min]**	17 [14 - 24]	14 [11 - 16]	19 [13 - 21]	13 [11 - 16]	18 [12 - 22]	13 [11 - 14]	17 [10 - 21]	14 [13 - 17]	16 [12 - 19]	13 [12 - 18]	16 [9 - 21]	13 [12 - 18]	15 [12 - 22]	17 [12 - 19]	18 [12 - 20]	15 [11 - 19]	16 [13 - 18]	11 [9 - 18]	< 0.0001	0.28

CV – Conventional Ventilation; CLV – Closed-Loop Ventilation; PEEP – Positive End-Expiratory Pressure

Supplemental Figure e1: Oxygenation parameters stratified by ventilation mode over the first week of ventilation. For ease of visualization, individual patient data was averaged into 6-hour intervals. Lines represent median values, shaded areas the interquartile ranges.

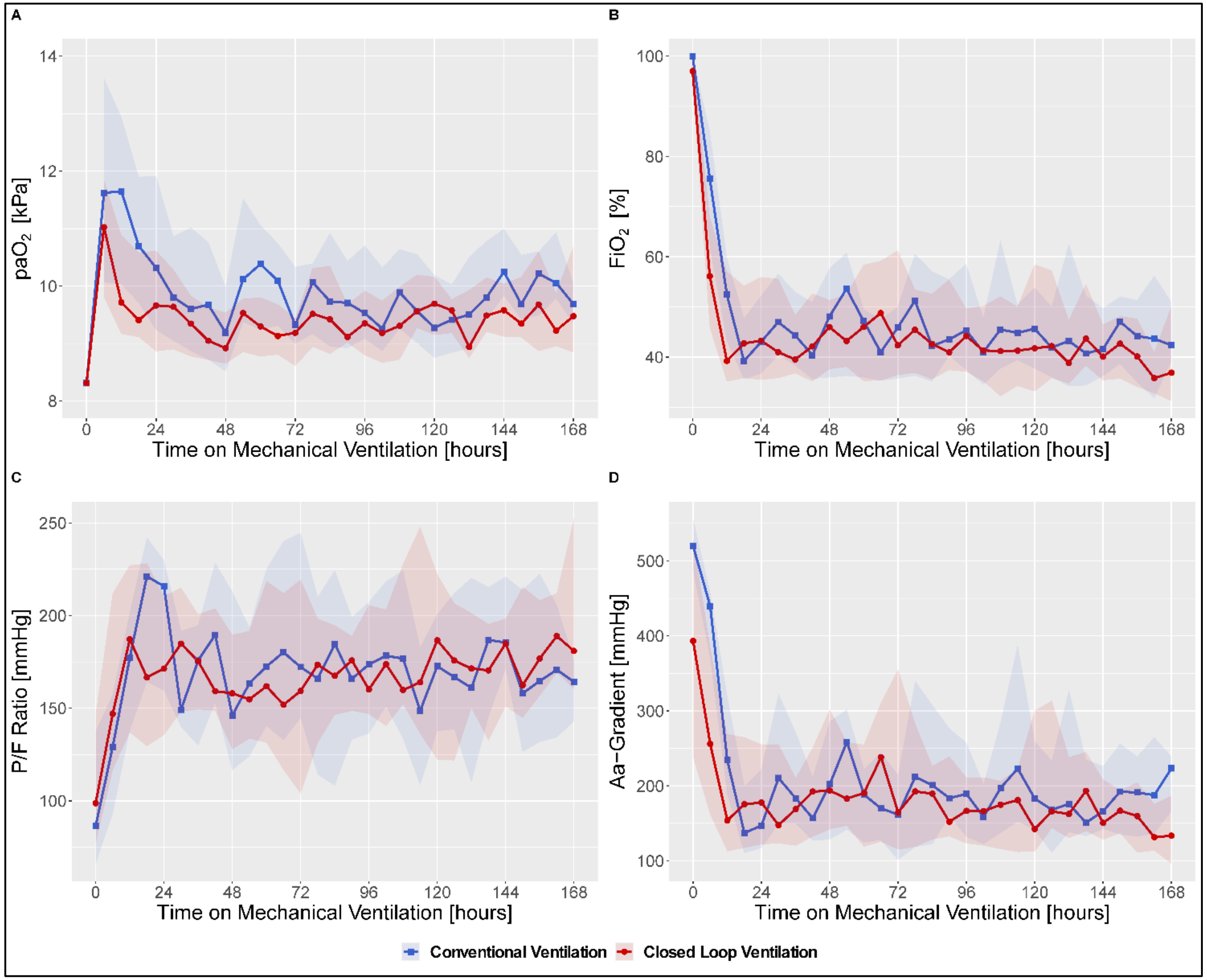

Supplemental Table e3: Mixed Effect Model of oxygenation parameters stratified by ventilation mode over the first week of ventilation.

	**0h - CV**	**0h - CLV**	**6h - CV**	**6h - CLV**	**12h - CV**	**12h - CLV**	**18h - CV**	**18h - CLV**	**24h - CV**	**24h - CLV**	**30h - CV**	**30h - CLV**	**36h - CV**	**36h - CLV**	**42h - CV**	**42h - CLV**	**48h - CV**	**48h - CLV**	**54h - CV**	**54h - CLV**
**paO_2 [kPa]**	8.3 [7.9 - 8.9]	8.3 [7.8 - 9.2]	11.6 [10.1 - 13.6]	11.0 [9.8 - 11.8]	11.7 [10.0 - 12.9]	9.7 [9.2 - 10.9]	10.7 [9.7 - 12]	9.4 [9.1 - 10.6]	10.3 [9.2 - 11.9]	9.7 [8.9 - 10.6]	9.8 [9.1 - 10.9]	9.6 [8.9 - 10.3]	9.6 [8.9 - 11.0]	9.4 [8.8 - 9.8]	9.7 [8.7 - 10.8]	9.1 [8.7 - 9.6]	9.2 [8.5 - 10.0]	8.9 [8.7 - 9.5]	10.1 [9.4 - 11.5]	9.5 [8.9 - 9.8]
**FiO_2 [%]**	100 [96 - 100]	97 [95 - 100]	76 [62 - 85]	56 [46 - 68]	52 [43 - 60]	39 [35 - 57]	39 [36 - 48]	43 [36 - 54]	43 [37 - 52]	43 [35 - 56]	47 [40 - 57]	41 [36 - 56]	44 [38 - 53]	40 [37 - 48]	40 [36 - 51]	42 [35 - 53]	48 [36 - 58]	46 [38 - 51]	54 [36 - 61]	43 [38 - 53]
**P/F Ratio [mmHg]**	86 [66 - 137]	99 [77 - 147]	129 [93 - 157]	147 [117 - 212]	177 [138 - 202]	187 [137 - 227]	221 [164 - 242]	167 [130 - 228]	216 [160 - 230]	171 [136 - 210]	149 [140 - 192]	185 [148 - 215]	176 [130 - 195]	175 [149 - 201]	190 [153 - 229]	159 [149 - 204]	146 [117 - 213]	158 [128 - 190]	163 [124 - 193]	155 [134 - 192]
**Aa-Gradient [mmHg]**	520 [488-556]	393 [238-530]	440 [371 - 458]	256 [162 - 384]	234 [189 - 319]	154 [113 - 269]	137 [110 - 200]	176 [117 - 265]	147 [117 - 223]	178 [122 - 255]	211 [150 - 322]	148 [123 - 255]	184 [150 - 272]	170 [120 - 210]	157 [127 - 212]	193 [132 - 251]	202 [129 - 286]	194 [142 - 302]	259 [142 - 302]	183 [147 - 258]

	60h - CV	60h - CLV	66h - CV	66h - CLV	72h - CV	72h - CLV	78h - CV	78h - CLV	84h - CV	84h - CLV	90h - CV	90h - CLV	96h - CV	96h - CLV	102h - CV	102h - CLV	108h - CV	108h - CLV	114h - CV	114h - CLV
**paO_2 [kPa]**	10.4 [9.2 - 11.1]	9.3 [8.8 - 9.8]	10.1 [9.3 - 10.7]	9.1 [8.8 - 9.7]	9.3 [9.0 - 10.3]	9.2 [8.6 - 9.4]	10.1 [9.2 - 10.4]	9.5 [8.9 - 10.3]	9.7 [9.0 - 10.9]	9.4 [8.8 - 10.4]	9.7 [8.9 - 10.4]	9.1 [9.0 - 9.7]	9.5 [9.1 - 10.7]	9.4 [8.8 - 9.9]	9.3 [8.9 - 10.3]	9.2 [8.7 - 9.8]	9.9 [9.3 - 10.6]	9.3 [8.7 - 10.0]	9.6 [9.0 - 10.6]	9.6 [9.3 - 10.2]
**FiO_2 [%]**	47 [36 - 50]	46 [35 - 58]	41 [35 - 48]	49 [35 - 59]	46 [36 - 50]	42 [36 - 61]	51 [36 - 61]	45 [37 - 53]	42 [35 - 57]	43 [36 - 53]	44 [40 - 56]	41 [37 - 55]	45 [37 - 59]	44 [37 - 50]	41 [35 - 48]	41 [35 - 50]	45 [38 - 63]	41 [32 - 52]	45 [39 - 50]	41 [34 - 50]
**P/F Ratio [mmHg]**	173 [141 - 225]	162 [131 - 219]	180 [133 - 240]	152 [117 - 212]	172 [145 - 245]	159 [104 - 219]	166 [114 - 210]	173 [131 - 198]	185 [108 - 224]	168 [146 - 195]	166 [133 - 199]	176 [149 - 189]	174 [136 - 207]	160 [147 - 206]	178 [151 - 218]	174 [140 - 203]	177 [134 - 224]	160 [152 - 228]	149 [109 - 183]	164 [136 - 248]
**Aa-Gradient [mmHg]**	188 [125 - 222]	191 [119 - 255]	170 [128 - 244]	238 [126 - 311]	162 [101 - 214]	165 [115 - 355]	212 [119 - 340]	193 [119 - 283]	201 [122 - 308]	190 [129 - 227]	184 [161 - 277]	152 [133 - 239]	189 [132 - 257]	167 [123 - 211]	159 [123 - 202]	166 [120 - 212]	197 [137 - 285]	175 [116 - 207]	223 [153 - 388]	181 [112 - 224]

	120h - CV	120h - CLV	126h - CV	126h - CLV	132h - CV	132h - CLV	138h - CV	138h - CLV	144h - CV	144h - CLV	150h - CV	150h - CLV	156h - CV	156h - CLV	162h - CV	162h - CLV	168h - CV	168h - CLV	p - Group	p - Time
**paO_2 [kPa]**	9.3 [8.8 - 10.2]	9.7 [9.2 - 10.2]	9.4 [8.8 - 10.0]	9.6 [9.2 - 9.8]	9.5 [8.9 - 10.5]	8.9 [8.8 - 9.9]	9.8 [9.4 - 10.8]	9.5 [9.2 - 10.2]	10.3 [9.8 – 11.0]	9.6 [9.1 - 10.0]	9.7 [9.4 - 10.5]	9.4 [9.1 - 10.2]	10.2 [9.5 - 10.6]	9.7 [8.9 - 10.6]	10.1 [9.8 - 10.9]	9.2 [9.0 - 9.9]	9.7 [9.4 - 10.0]	9.5 [8.9 - 10.7]	0.05	0.25
**FiO_2 [%]**	46 [38 - 54]	42 [33 - 58]	42 [36 - 50]	42 [36 - 57]	43 [34 - 63]	39 [35 - 47]	41 [34 - 52]	44 [38 - 55]	42 [36 - 50]	40 [35 - 46]	47 [39 - 52]	43 [36 - 48]	44 [35 - 51]	40 [34 - 48]	44 [32 - 56]	36 [33 - 41]	42 [39 - 51]	37 [31 - 50]	0.88	< 0.0001
**P/F Ratio [mmHg]**	173 [138 - 200]	187 [122 - 222]	167 [139 - 212]	176 [122 - 207]	161 [110 - 220]	171 [150 - 201]	187 [145 - 215]	170 [133 - 195]	185 [171 - 221]	185 [151 - 198]	158 [126 - 214]	162 [145 - 215]	165 [132 - 223]	177 [157 - 208]	171 [134 - 206]	189 [169 - 212]	164 [143 - 176]	181 [161 - 252]	0.04	0.4
**Aa-Gradient [mmHg]**	183 [142 – 259]	142 [112 - 300]	168 [111 - 233]	166 [144 - 314]	176 [139 - 327]	163 [130 - 238]	151 [133 - 235]	193 [140 - 245]	166 [143 - 226]	1501 [128 - 208]	192 [135 - 256]	167 [137 - 194]	191 [132 - 239]	160 [111 - 204]	188 [137 - 265]	131 [114 - 176]	223 [166 - 239]	134 [96 - 187]	0.04	0.3

CV – Conventional Ventilation; CLV – Closed-Loop Ventilation; paO_2 – partial pressure of arterial oxygen; FiO_2 – Fraction of inspired oxygen; A-a Gradient – Alveolar-arterial Gradient

Supplemental Figure e2: Ventilation parameters stratified by ventilation mode over the first week of ventilation. For ease of visualization, individual patient data was averaged into 6-hour intervals. Lines represent median values, shaded areas the interquartile ranges.

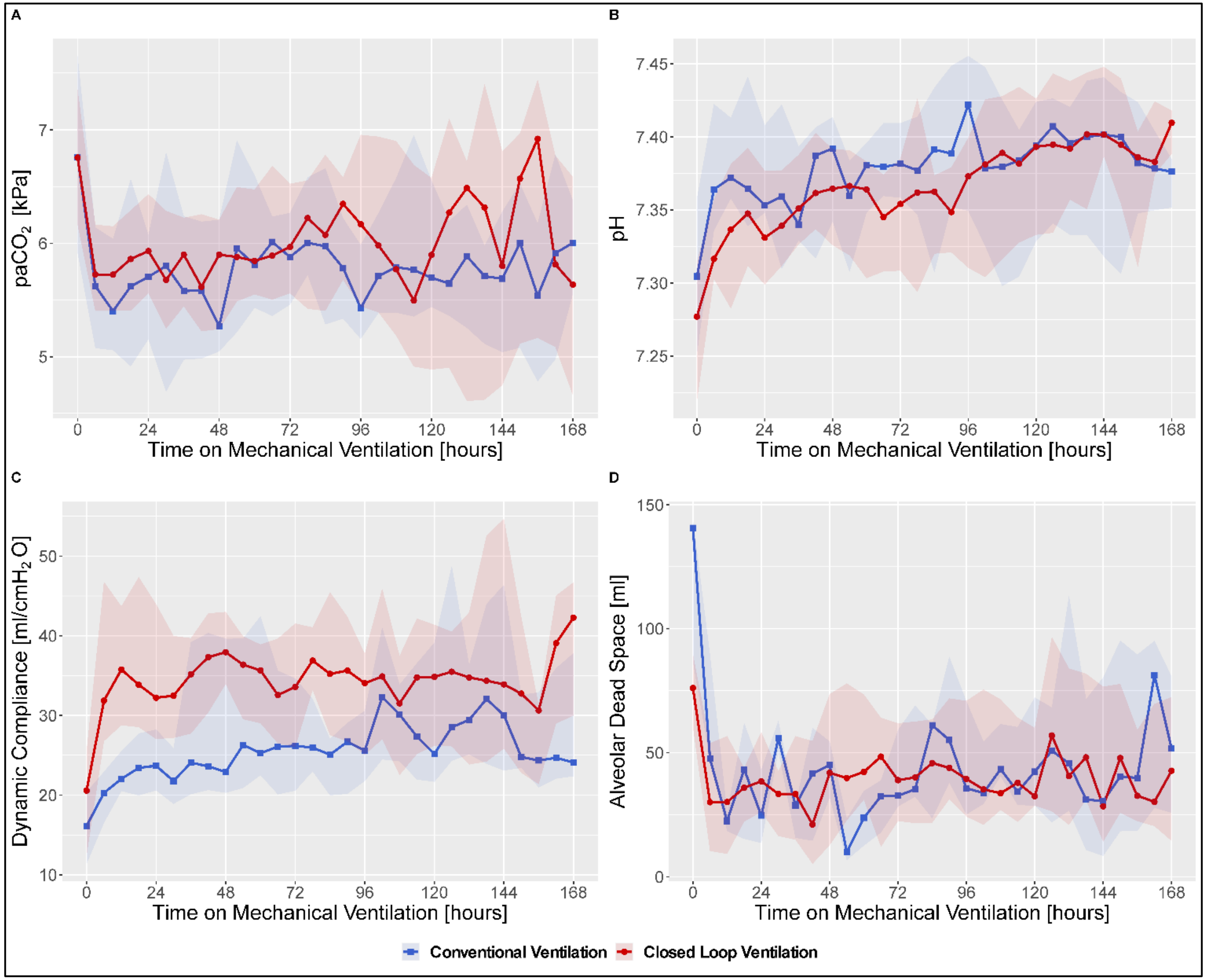

Supplemental Table e4: Mixed Effect Model of ventilation parameters stratified by ventilation mode over the first week of ventilation.

	0h - CV	0h - CLV	6h - CV	6h - CLV	12h - CV	12h - CLV	18h - CV	18h - CLV	24h - CV	24h - CLV	30h - CV	30h - CLV	36h - CV	36h - CLV	42h - CV	42h - CLV	48h - CV	48h - CLV	54h - CV	54h - CLV
**$paCO_2$ [kPa]**	6.8 [5.9 - 7.7]	6.8 [6.2 - 7.4]	5.6 [5.1 - 6.1]	5.7 [5.4 - 6.2]	5.4 [5.1 - 6.0]	5.7 [5.4 - 6.2]	5.6 [4.9 - 6.6]	5.9 [5.4 - 6.3]	5.7 [5.2 - 6.1]	5.9 [5.6 - 6.4]	5.8 [4.7 - 6.8]	5.7 [5.3 - 6.3]	5.6 [5.0 - 6.2]	5.9 [5.5 - 6.2]	5.6 [5.0 - 6.2]	5.6 [5.2 - 6.3]	5.3 [5.1 - 6.2]	5.9 [5.2 - 6.2]	6.0 [5.2 - 6.9]	5.9 [5.5 - 6.5]
**pH**	7.30 [7.25 - 7.36]	7.28 [7.22 - 7.31]	7.36 [7.34 - 7.42]	7.32 [7.3 - 7.36]	7.37 [7.36 - 7.41]	7.34 [7.28 - 7.38]	7.36 [7.34 - 7.44]	7.35 [7.31 - 7.39]	7.35 [7.35 - 7.41]	7.33 [7.3 - 7.38]	7.36 [7.31 - 7.42]	7.34 [7.31 - 7.38]	7.34 [7.33 - 7.39]	7.35 [7.33 - 7.39]	7.39 [7.32 - 7.41]	7.36 [7.34 - 7.4]	7.39 [7.34 - 7.41]	7.36 [7.33 - 7.39]	7.36 [7.31 - 7.38]	7.37 [7.32 - 7.39]
**Dynamic Compliance [ml/ cmH_2O]**	16 [11 - 20]	21 [12 - 26]	20 [17 - 23]	32 [27 - 47]	22 [21 - 25]	36 [29 - 44]	23 [20 - 28]	34 [29 - 47]	24 [21 - 28]	32 [27 - 44]	22 [19 - 26]	32 [27 - 40]	24 [20 - 39]	35 [28 - 40]	24 [21 - 40]	37 [30 - 43]	23 [20 - 40]	38 [34 - 43]	26 [21 - 40]	36 [30 - 40]
**Alveolar Dead Space [ml]**	141 [87- 144]	76 [61- 90]	48 [47 - 89]	30 [10 - 54]	22 [18 - 26]	30 [9 - 57]	43 [15 - 62]	36 [22 - 44]	25 [14 - 43]	39 [26 - 58]	56 [51 - 63]	33 [16 - 43]	29 [16 - 29]	33 [15 - 57]	42 [15 - 60]	21 [5.2 - 55]	45 [32 - 55]	41 [13 - 74]	10 [7 - 23]	40 [23 - 78]

	**60h - CV**	**60h - CLV**	**66h - CV**	**66h - CLV**	**72h - CV**	**72h - CLV**	**78h - CV**	**78h - CLV**	**84h - CV**	**84h - CLV**	**90h - CV**	**90h - CLV**	**96h - CV**	**96h - CLV**	**102h - CV**	**102h - CLV**	**108h - CV**	**108h - CLV**	**114h - CV**	**114h - CLV**
**paCO_2 [kPa]**	5.8 [5.4 - 6.6]	5.9 [5.6 - 6.5]	6.0 [5.4 - 6.2]	5.9 [5.5 - 6.7]	5.9 [5.5 - 6.3]	6.0 [5.6 - 6.5]	6.0 [5.7 - 6.6]	6.2 [5.4 - 6.6]	6.0 [5.3 - 6.7]	6.1 [5.4 - 6.8]	5.8 [5.3 - 6.2]	6.4 [5.7 - 6.6]	5.4 [5.2 – 6.0]	6.2 [5.4 – 7.0]	5.7 [5.4 - 6.3]	6.0 [5.4 - 6.9]	5.8 [5.4 - 6.6]	5.8 [5.2 - 6.9]	5.8 [5.4 – 7.0]	5.5 [4.9 - 6.7]
**pH**	7.38 [7.35 - 7.4]	7.36 [7.3 - 7.38]	7.38 [7.36 - 7.41]	7.35 [7.31 - 7.37]	7.38 [7.36 - 7.41]	7.35 [7.31 - 7.39]	7.38 [7.35 - 7.41]	7.36 [7.29 - 7.43]	7.39 [7.31 - 7.43]	7.36 [7.32 - 7.37]	7.39 [7.35 - 7.45]	7.35 [7.32 - 7.38]	7.42 [7.35 - 7.46]	7.37 [7.31 - 7.4]	7.38 [7.33 - 7.45]	7.38 [7.3 - 7.43]	7.38 [7.3 - 7.43]	7.39 [7.32 - 7.43]	7.38 [7.3 - 7.4]	7.38 [7.33 - 7.43]
**Dynamic Compliance [ml/ cmH_2O]**	25 [23 - 42]	36 [29 - 39]	33 [18 – 39]	48 [12 - 64]	33 [28 - 59]	39 [22 - 62]	35 [32 - 69]	40 [22 - 63]	61 [24 - 61]	46 [22 - 63]	55 [38 - 89]	44 [31 - 72]	36 [25 - 71]	39 [24 - 71]	34 [29 - 54]	35 [21 - 75]	43 [41 - 62]	34 [27 - 71]	34 [29 - 60]	38 [22 - 64]
**Alveolar Dead Space [ml]**	24 [13 - 35]	42 [31 - 73]	26 [20 - 36]	33 [27 - 40]	26 [21 - 35]	34 [26 - 42]	26 [23 - 32]	37 [33 - 41]	25 [20 - 27]	35 [28 - 45]	27 [20 - 29]	36 [27 - 42]	26 [22 - 31]	34 [25 - 38]	32 [24 - 41]	35 [27 - 46]	30 [24 - 34]	32 [23 - 37]	27 [22 - 37]	35 [25 - 42]

	120h - CV	120h - CLV	126h - CV	126h - CLV	132h - CV	132h - CLV	138h - CV	138h - CLV	144h - CV	144h - CLV	150h - CV	150h - CLV	156h - CV	156h - CLV	162h - CV	162h - CLV	168h - CV	168h - CLV	p - Group	p - Time
**$paCO_2$ [kPa]**	5.7 [5.4 - 6.6]	5.9 [4.9 - 6.6]	5.7 [5.4 - 6.2]	6.3 [4.9 - 7.1]	5.9 [5.3 - 6.3]	6.5 [4.6 - 6.7]	5.7 [5.1 – 6.0]	6.3 [4.6 - 7.4]	5.7 [5.0 - 6.3]	5.8 [4.8 - 6.8]	6.0 [5.1 - 6.5]	6.6 [5.1 – 7.0]	5.5 [4.8 - 6.2]	6.9 [5.2 - 7.4]	5.9 [5.0 - 6.8]	5.8 [5.1 - 6.8]	6.0 [5.6 - 6.4]	5.6 [4.7 - 6.6]	0.41	0.8
**pH**	7.39 [7.33 - 7.42]	7.39 [7.33 - 7.43]	7.41 [7.34 - 7.43]	7.39 [7.33 - 7.44]	7.4 [7.33 - 7.42]	7.39 [7.36 - 7.44]	7.4 [7.33 - 7.44]	7.4 [7.35 - 7.44]	7.4 [7.32 - 7.44]	7.4 [7.39 - 7.45]	7.4 [7.31 - 7.43]	7.39 [7.35 - 7.44]	7.38 [7.35 - 7.42]	7.39 [7.31 - 7.4]	7.38 [7.35 - 7.4]	7.38 [7.37 - 7.42]	7.38 [7.35 - 7.38]	7.41 [7.39 - 7.42]	0.68	< 0.0001
**Dynamic Compliance [ml/ cmH_2O]**	42 [29 - 72]	32 [29 - 60]	51 [22 - 68]	57 [26 - 97]	46 [27 - 113]	41 [21 - 84]	31 [11 - 72]	48 [31 - 82]	30 [8.- 80]	28 [14 - 76]	40 [18 - 95]	48 [26 - 78]	40 [19 - 90]	33 [22 - 59]	81 [28 - 95]	30 [20 - 70]	52 [26 - 81]	43 [15 - 72]	< 0.01	0.15
**Alveolar Dead Space [ml]**	25 [22 - 39]	35 [26 - 41]	29 [24 - 49]	35 [26 - 41]	29 [25 - 36]	35 [24 - 43]	32 [24 - 44]	34 [24 - 53]	30 [23 - 46]	34 [26 - 55]	25 [23 - 33]	33 [23 - 42]	24 [21 - 33]	31 [21 - 31]	25 [22 - 36]	39 [29 - 45]	24 [22 - 38]	42 [30 - 47]	< 0.01	0.1

CV – Conventional Ventilation; CLV – Closed-Loop Ventilation; $paCO_2$ – Partial pressure of arterial carbon dioxide

Supplemental Table e5: Percentage of time of lung-protective mechanical ventilation during the first 7 days of mechanical ventilation

	Overall Population N =40	Conventional Ventilation N = 17	Closed Loop Ventilation N = 23	p
**n [minutes]**	348319	173626	174693	
**Tidal Volume/ IBW >10 ml/ kg**	4985 (1.5)	1473 (1.0)	3512 (2.0)	<0.001
**Tidal Volume/ IBW 8-10 ml/ kg**	17996 (5.5)	9368 (6.1)	8628 (4.9)	<0.001
**Tidal Volume/ IBW 6-8 ml/ kg**	107125 (32.7)	49105 (32.2)	58020 (33.2)	<0.001
**Tidal Volume/ IBW <6 ml/ kg**	197323 (60.3)	92791 (60.8)	104532 (59.8)	<0.001
**Dynamic Driving Pressure <15 cmH$_2$O**	223433 (68.5)	76518 (50.5)	146915 (84.1)	<0.001
**Peak Inspiratory Pressure <30 cmH$_2$O**	265639 (81.1)	109763 (71.9)	155876 (89.2)	<0.001
**Dynamic Mechanical Power <17 J/min**	213639 (65.5)	76351 (50.4)	137288 (78.6)	<0.001
**paO$_2$ >7.33kPa**	2982 (98.4)	1462 (97.9)	1520 (98.8)	0.067
**SpO$_2$ >88%**	331704 (97.1)	162598 (96.7)	169106 (97.5)	<0.001
**pH >7.25**	2851 (92.8)	1380 (91.9)	1471 (93.7)	0.069
**Full Lung Protectedness †**	170471 (52.3)	56788 (37.5)	113683 (65.1)	<0.001

paO$_2$ – partial pressure of arterial oxygen; SpO$_2$ – Peripheral oxygen saturation; FiO$_2$ – Fraction of inspired oxygen; PEEP – Positive End-Expiratory Pressure; IBW – Ideal Body Weight. † Full Lung Protectiveness was defined as the conjoined target of tidal volumes <8 ml/ kg, peak inspiratory pressure <30 cmH$_2$O, dynamic driving pressure <15 cmH$_2$O, peripheral oxygen saturation >88 % and a dynamic mechanical power <17 J/min.

Supplemental Table e6: Percentage of time of lung-protective mechanical ventilation during the overall time of mechanical ventilation

	Overall Population N =40	Conventional Ventilation N = 17	Closed Loop Ventilation N = 23	p
**n [minutes]**	1048630	415047	633583	
**Tidal Volume/ IBW >10 ml/ kg**	19568 (1.9)	8324 (2.2)	11244 (1.8)	<0.001
**Tidal Volume/ IBW 8-10 ml/ kg**	83302 (8.2)	38236 (9.9)	45066 (7.1)	<0.001
**Tidal Volume/ IBW 6-8 ml/ kg**	337816 (33.1)	121013 (31.3)	216803 (34.2)	<0.001
**Tidal Volume/ IBW <6 ml/ kg**	579987 (56.8)	219536 (56.7)	360451 (56.9)	0.074
**Dynamic Driving Pressure <15 cmH_2O**	605309 (59.4)	190100 (49.3)	415209 (65.5)	<0.001
**Peak Inspiratory Pressure <30 cmH_2O**	852199 (83.5)	293415 (75.8)	558784 (88.2)	<0.001
**Dynamic Mechanical Power <17 J/min**	643090 (63.1)	214894 (55.8)	428196 (67.6)	<0.001
**paO_2 >7.33kPa**	8620 (98.6)	3513 (98.3)	5107 (98.8)	0.038
**SpO_2 >88%**	1007213 (97.8)	390882 (97.2)	616331 (98.2)	<0.001
**pH >7.25**	8476 (95.3)	3371 (93.0)	5105 (96.9)	<0.001
**Full Lung Protectedness †**	414278 (40.7)	127983 (33.2)	286295 (45.2)	<0.001

paO_2 – partial pressure of arterial oxygen; SpO_2 – Peripheral oxygen saturation; FiO_2 – Fraction of inspired oxygen; PEEP – Positive End-Expiratory Pressure; IBW – Ideal Body Weight. † Full Lung Protectiveness was defined as the conjoined target of tidal volumes <8 ml/ kg, peak inspiratory pressure <30 cmH_2O, dynamic driving pressure <15 cmH_2O, peripheral oxygen saturation >88 % and a dynamic mechanical power <17 J/min.
